# Investigation of genetic diversity and polyandry of *Leptinotarsa decemlineata* using X-linked microsatellite markers

**DOI:** 10.1038/s41598-023-49002-7

**Published:** 2023-12-11

**Authors:** P. Sedlák, V. Sedláková, J. Vašek, M. Melounová, D. Čílová, P. Vejl, O. Skoková Habuštová, P. Doležal, E. Hausvater

**Affiliations:** 1 Department of Genetics and Breeding, Faculty of Agrobiology Food and Natural Resources, Czech University of Life Sciences Prague, Kamýcká 129, 16500 Prague 6, Suchdol Czech Republic; 2 Biology Centre, Institute of Entomology, Czech Academy of Sciences, Branišovská 1160/31, 37005 České Budějovice, Czech Republic; 3 Department of Potato Protection, Potato Research Institute Havlíčkův Brod. Ltd., Dobrovského 2366, 58001 Havlíčkův Brod, Czech Republic

**Keywords:** Genetic variation, Entomology, Invasive species, Sexual selection

## Abstract

A panel of X-linked microsatellite markers was newly designed using the data from a previous sequencing project available in NCBI and used for a study of the Colorado potato beetle (CPB, *Leptinotarsa decemlineata*) X-haplotype variability. The analysis of scaffolds 49 and 61 (newly identified as fragments of CPB chromosome X) found ten high-quality markers, which were arranged in two PCR multiplexes and evaluated in both 420 CPB adults, collected from 14 localities of Czechia and Slovakia, and 866 larvae from five single-female families from two more Czech localities. Length polymorphisms found in 6 loci have predicted 192 potential X-haplotypes, however, only 36 combinations were detected in the adult males (N = 189), and seven additional ones in the larvae. The X-haplotypes were also generally unevenly distributed; five of the most frequent haplotypes were detected in 55% of males, 19 repeating up to ten-times in 38.7% of males and the remained 12 occurred uniquely in 6.3% of males. Bulk analysis of X-haplotypes dissimilarity indicated seven haplotype groups diversified by mutations and recombinations. Two haplotypes showed a distinctive regional distribution, which indicates an east–west disruption of CPB migration probably caused by different environments of localities in the South Bohemia region and Vysocina region. On the contrary, the results indicate a south–north migration corridor alongside the Vltava River. In the single-female families, from 6 to 13 distinct paternal haplotypes were detected, which proved and quantified a frequented polyandry in CPB.

## Introduction

The Colorado potato beetle (CPB, *Leptinotarsa decemlineata* Say.) is an invasive species of economic importance worldwide^1–3^. The invasion of CPB to Europe represents a typical example of population expansion under the effect of a bottleneck selection^2,4,5^. This is when the population expands quickly despite a limited genetic diversity from a few progenitors. Probably due to an adaptation ability, the species, facing the dynamic development of agricultural technologies, chemical protection, and other environmental changes, has quickly become the most destructive pest of solanaceous crops in Eurasia.

In the past, the complex research on CPB has uncovered extensive information from an important biological, ecological, and genetic characteristic. Particularly regarding the adaptation and reproduction mechanisms beneficial to the invasiveness and destructiveness of CPB^6,7^. The first key biological and genetic characteristics of CPB identified, were the karyotype 2n = 32 + X0 or 34 + X0^8^ and X0 sex determination system^9,10^. Studies based on isozymes markers introduced knowledge of CPB reproduction strategy, particularly the discovery of a potential for polygynandry^11^ and a last-male precedence^12^. The introduction of standard approaches to DNA analysis enabled a study of polymorphisms in mtDNA^2^ and microsatellite loci^13^. Subsequent extensive comparative population research confirmed the genetic distinctness of CPB populations in Northern America and Europe^2,13^. Later, the distinctness and dynamics were reported for Asian CPB populations^14^, where the invasion started only several years ago^1^. Basic genetic research has continued in advanced sequencing projects. While the first project^15^ identified many valuable, though not yet assembled, contigs of CPB genome sequences, the most recent project^16^ identified the complete sequences of all chromosomes.

The application of molecular studies in agronomic research uncovered some principles and mechanisms responsible for resistance to active compounds of insecticides. This has enriched the potential of population studies and has been reflected in dynamic changes of pest management strategies. The mapping of the *Ldvssc* gene related to *kdr* resistance to pyrethroids on X-chromosome^17–19^ initiated the recent development of method for sexing of larvae on a principle of copy number variation (CNV) of the X-linked genes^20^. This later helped confirm a general shift in the primary sex-ratio in a favour of females^21^. By using the microsatellite markers, the study also confirmed polygynandry as a standard in the reproductive strategy of CPB. The real involvement of various males in the family structure, yet unresolved, was also discussed.

The objective of this paper is to present the results of research aimed at the design and subsequent use of a new protocol of CPB X-chromosome haplotype analysis, which hypothesises that a specific genetic structure indicative for population dynamics can be found, and that different male X-haplotypes can be detected with relative precision against the background of maternal haplotypes in the half-siblings to explain the reproductive behaviour of CPB.

## Material and methods

### Consent with collections

Adults of CPB were collected with consent from the owners of experimental and production potato fields.

### Biological material

In June of 2019, a sample of 1,400 overwintering adults of CPB was collected in 13 potato growing areas of the Czech Republic and in one distant locality of Slovakia. In each locality, the adults were sampled randomly immediately after they occurred in potato fields. 100 adults per hectare (approximately one random beetle per 10 m^2^) were collected to prevent the genetic relations of individuals. After collection, the adults were quickly frozen in liquid nitrogen and kept for further processing at − 20 °C.

**Figure 1:**
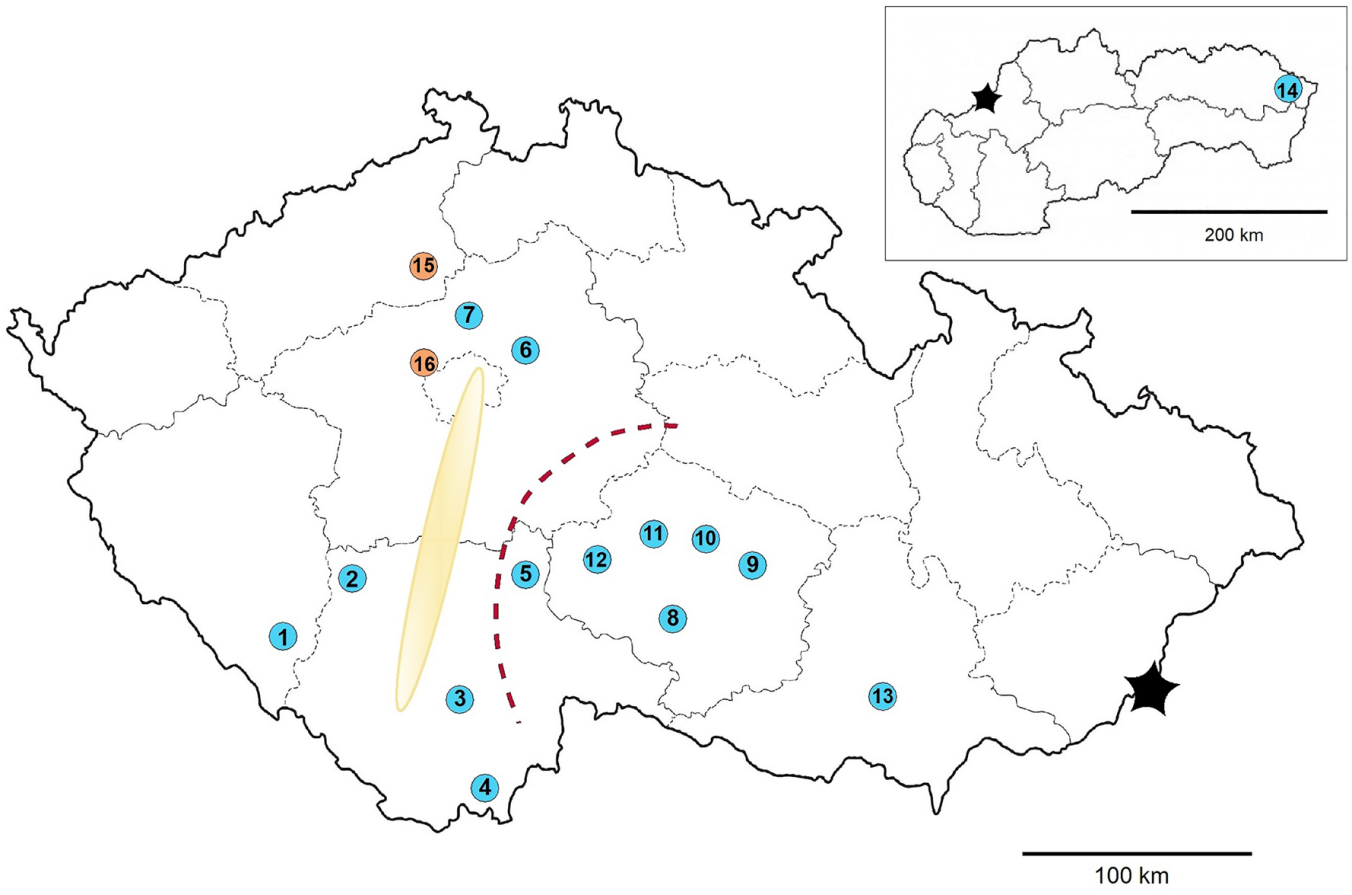
The sites of collections of the CPB adults (blue): 1) Bojanovice, 2) Belcice, 3) Ceske Budejovice, 4) Malonty, 5) Oblajovice, 6) Celakovice, 7) Chlumin, 8) Cizov, 9) Havlickova Borova, 10) Jamy, 11) Valecov, 12) Zeliv, 13) Zabcice, 14) Stakcin (Slovakia). The sites of collections of the mothers of clutches (orange): 15) Travcice, 16) Prague 6 Ruzyne. Relatively to the results, the yellow ellipse indicates the open migration corridor, the red line represents the potential barrier of migration.

In parallel, an additional set of randomly fertilised females was collected in two other localities to obtain single-female families of 1st instar larvae under laboratory conditions, from which five families (TR10, TR11, TR12, TR14 and RU14) were chosen for the study of polyandry. After hatching, the larvae were immediately individually placed into tubes, frozen in liquid nitrogen and stored for next processing at − 20 °C. The mothers were processed the same way as other adults after oviposition. The method of temporary lab cultivations, processing and sexing of larvae was presented in detail previously by Sedlakova et al.^21^. The collections and their geographical distribution are presented in Table 1 and Fig. 1.

**Table 1:** Overview of sites of CPB collections and their structure in terms of sex-ratios.

Locality No:	Locality	Region	N	N_males_	N_females_
	*Field collections of CPB adults*		*420*	*189*	*231*
1	Bojanovice	Plzensky	30	15	15
2	Belcice	South Bohemia	30	15	15
3	Ceske Budejovice	South Bohemia	30	12	18
4	Malonty	South Bohemia	30	11	19
5	Oblajovice	South Bohemia	30	10	20
6	Celakovice	Central Bohemia	30	15	15
7	Chlumin	Central Bohemia	30	13	17
8	Cizov	Vysocina	30	11	19
9	Havlickova Borova	Vysocina	30	16	14
10	Jamy	Vysocina	30	15	15
11	Valecov	Vysocina	30	14	16
12	Zeliv	Vysocina	30	14	16
13	Zabcice	South Moravia	30	10	20
14	Stakcin	Presovsky (Slovakia)	30	18	12
	*Randomly fertilised mothers of larvae*		*866*	*362*	*504*
15	Travcice 10 (TR10)	Central Bohemia	127	48	79
	Travcice 11 (TR11)	Central Bohemia	253	117	136
	Travcice 12 (TR12)	Central Bohemia	206	77	129
	Travcice 14 (TR14)	Central Bohemia	205	91	114
16	Prague 6 Ruzyne 14 (RU14)	Central Bohemia	75	29	46

A general bias of sex-ratio to the benefit of females is apparent.

### Processing of samples and DNA extractions

Thirty random individuals from each set of adults were chosen and sexed using morphological markers by Khidhir and Mustafa^22^. To obtain genomic DNA, three legs of each adult were placed into 0.5 ml polypropylene tube and extracted using the protocol published by Sedlakova et al.^20^. Genomic DNA from entire individual larvae was extracted the same way. Pure DNA samples were quantified using Nanophotometer (Implen, Germany) and diluted to 5 ng.ml^−1^ by 1xTE buffer.

### Design of primers

**Figure 2:**
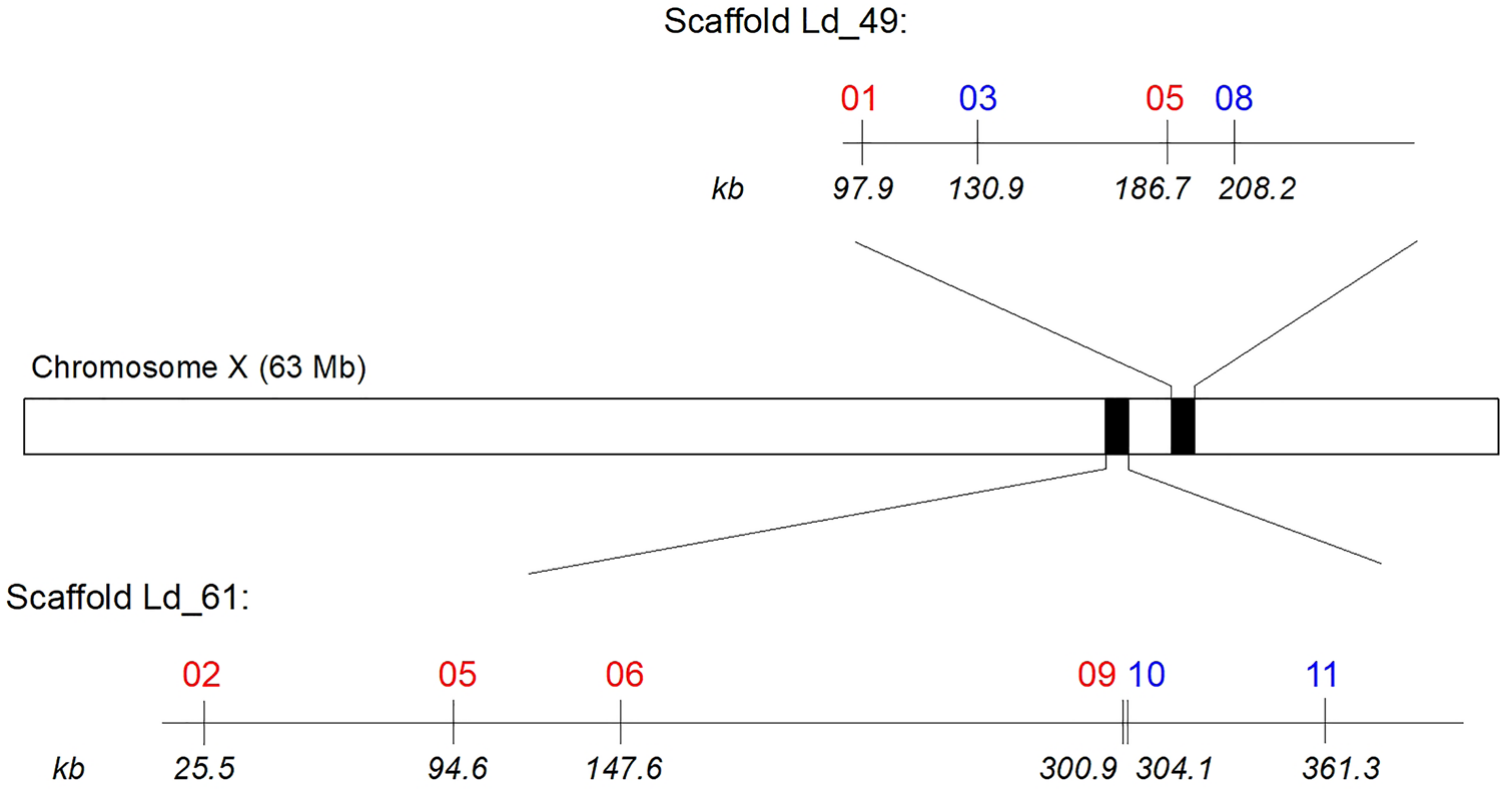
Localisation of microsatellites in the scaffolds 49 (KZ312502.1) and 61 (KZ312483.1), and approximate localisation of scaffolds in the CPB chromosome X. The red text was used to highlight the polymorphic loci.

The primers were designed based on scaffolds 49 (KZ312502.1) and 61 (KZ312483.1) from CPB whole genome shotgun sequencing project available in GenBank NCBI, published by i5K Consortium^23^. The scaffolds were identified as candidates on X-chromosome using homology with *Tribolium castaneum* (Herbst, 1797) genome published by Richards et al.^24^ in several rounds of cross-validation. Firstly, all 744 X chromosomal genes of the red flour beetle were checked and mRNA sequences of ~ 100 protein coding genes were BLASTed^25^ against CPB transcriptome. Then the individual mRNA transcripts were paired with the loci of given scaffold. This preliminary research led to the identification of 60 unique scaffolds with presumed X chromosomal origin and the most promising ones (21) were further analysed. In the next step, mRNA sequences of all genes of the given scaffold (~ 10 genes per scaffold on average) were BLASTed against *Tribolium* genome, which increased the chance that the given scaffold truly belonged to CPB X chromosome; only scaffolds with most of the genes linked to the *Tribolium* linkage group LG1 (chromosome X) were used for downstream analyses. Finally, 2 out of 11 remaining scaffolds were selected and exploited for SSR panel design. Within the selected scaffolds, the microsatellite loci were searched using GMATA v2.0^26^ with the following parameters: motif length 3–6 bp, the minimum number of repetitions 5, and amplicon size range 100–400 bp. Single copy occurrence of selected loci was verified against the CPB genome and locus specific primers were designed using Primer3web v4.1.0^27,28^ and OligoEvaluator (an online tool provided by Merck company—http://www.oligoevaluator.com/LoginServlet). A localisation of microsatellites on the scaffolds is presented in Fig. 2, exact positions on the X-chromosome are presented in Table 2.

**Table 2:** Characteristics of markers.

Locus	Position on chr. X (bp)	Primer sequence (5’–3’) and 5’ label	T_m_	Conc [µM]	Motif	Allele (bp)
	*Multiplex A*					
Ld4901	56,217,757 56,217,907	f: PET-ACACAATCATTCTAGCGCCG r: TTGGAAGCATAATCGTGGCAG	57.3 63.7	0.08 0.08	(TAA)_7_	144–147
Ld4903	56,256,738 56,256,948	f: 6-FAM-ACTGTAGAATCGCGGCTATGA r: TCATTGTTTTATGTTTCCTGCGT	57.9 61.3	0.04 0.04	(AAC)_5_	214
Ld4905	56,323,276 56,323,575	f: VIC-GCGTCTTAGATGTCAATGCCT r: CATATCTGATCGAGGAAGGTGT	57.9 63.9	0.04 0.04	(TTCA)_5_	295–300
Ld4908	56,350,983 56,351,137	f: 6-FAM-GCACGAACTCTTCAAACGGT r: TGCCATTTCGCCCTTTCAT	57.3 61.6	0.04 0.04	(AAT)_5_	155
Ld6111	52,920,157 52,920,335	f: NED-AAAGTTGAACGTGATGGTTGG r: TCCGTTGGCTTTGAAACCT	55.9 61.6	0.08 0.08	(TAT)_5_	174
	*Multiplex B*					
Ld6102	52,524,306 52,524,557	f: 6-FAM-TCACACCAATAGCTGCAATGA r: CAGGAGGATAGCACCAGTGA	55.9 65.0	0.08 0.08	(ACTC)_8_	244–252
Ld6105	52,598,946 52,599,155	f: PET-CCTGCATCGATTCCTTGTG r: CCACACAAACCTCTGACATATCT	56.7 64.0	0.08 0.08	(TGG)_7_	208–211
Ld6106	52,659,499 52,659,746	f: NED-CCCCTTTTCCGCTAATCTTC r: ACCGGTGGATGACAATATTCA	57.3 62.2	0.08 0.08	(ATT)_6_	236–242
Ld6109	52,841,444 52,841,758	f: VIC-TTTTAACACAGCCGGACGAT r: GGCTCCCACAAGAAATACAGA	55.3 63.7	0.08 0.08	(CTTAC)_5_	309–319
Ld6110	52,845,079 52,845,314	f: VIC-GAAGCGCAATTGAATGATGAC r: TTTGGAGCCACTTCTGTTTACT	55.9 62.4	0.04 0.04	(TAT)_5_	232

The SSR loci were subsequently amplified using a single-plex PCR and examined by Genetic Analyzer ABI PRISM 310. The PCR and separation conditions were the same as the conditions described for multiplex analysis below. The specificity of primers was verified via bidirectional sequencing of PCR products. The amplicons of each allele was sequenced twice in two biological replicates (adult males) originated from distant localities; however, three individuals were used to check the alleles of Ld4905, which expressed irregular, single-nucleotide, length polymorphisms. The amplicons of alleles were separated in 1% (w/v) agarose gel, excised after 30 min, and extracted by GeneJet Gel Extraction Kit (Thermo Fisher Scientific). After quantification by nanophotometer S-111107AW (Implen, Germany), the DNA samples were diluted as required by a sequencing service provider and subsequently sequenced (Eurofins Genomics Germany GmbH, Germany). The sequences were compared with the standard sequences of scaffolds and then submitted to NCBI nucleotide database.

### Multiplex analysis of SSR linked on X-chromosome

After the validations of markers, 10 primer pairs were arranged into two final multiplexes as described in Table 2. The PCR mix (10 µl) for both multiplexes contained 5 ng of genomic DNA, 5 µl of 2 × Multiplex PCR Master Kit (Qiagen, Germany) and all primers in recommended concentration (Table 2). PCR was performed in a C1000 thermocycler (BioRad, USA) using the program recommended by the multiplex kit provider with an optimised annealing temperature (60 °C). The amplicons were subsequently diluted 30 folds by PCR grade water and one µl of the diluted product was mixed with 12 µl of HiDi formamide (Applied Biosystems, USA) with 0.2 µl of size standard GeneScan LIZ600 (Applied Biosystems, USA). After denaturation (95 °C, 5 min), the amplicons were separated using Genetic Analyzer ABI PRISM 310 (Applied Biosystems, USA). Length polymorphisms were detected using the GeneMapper v 4.1 software (Applied Biosystems, USA).

### Detection of X-haplotypes, data processing and statistical evaluations

The dataset of alleles organized by sex, locality and region were analysed using GenAlEx 6.5 software^29,30^. The general descriptive population characteristics of loci were estimated for single loci and population samples of adults sorted differentially by sex (Tables 3 and 4). Genetic differentiation of sub-populations was evaluated using regional AMOVA with 999 bootstraps. This was also performed in GenAlEx 6.5, where the codominant diploid model (F-value) was used for females and haploid model (Φ-value) was used for males (Table 5). The polymorphic information content (PIC) was estimated using Gene-Calc^31^ and the potential risk of recent bottleneck effect was tested using Bottleneck version 1.2.02^32^, where the Wilcoxon test of “two-phase model (TPM)” with 95% participation of “strict one-step mutation model (SMM)” and variation value for TPM = 12 was used. Having one X-chromosome, the adult males were analysed to obtain information about the structure and frequencies of X-haplotypes using GenAlEx 6.5, however, the broader analysis and graphical comparison of male X-haplotypes was performed using an unweighted neighbour joining of dissimilarity coefficients in Darwin 6.0^33^. In the clutches produced by females TR10-RU14, the maternal X-haplotypes and their recombinants were identified using the X-haplotypes of male-larvae, whilst the genotypes of female-larvae were used for detection of paternal X-haplotypes. These analyses were performed individually with help of filters in MS Excel 2019 (Microsoft, USA). The figures and graphics were created using R-software^34^ and Zoner Callisto 5 (Zoner, Czech).

**Table 3:** Estimates of genetic diversity parameters by locus (mean ± SD) in 14 populations.

Locus							
*Males*	N_a_	N_e_	I	H	–	–	–
LdX-49–01	1.93 ± 0.07	1.49 ± 0.07	0.47 ± 0.05	0.31 ± 0.03			
LdX-49–03	1.0 ± 0.0	NA	NA	NA			
LdX-49–05	3.36 ± 0.13	2.51 ± 0.13	1.01 ± 0.04	0.59 ± 0.02			
LdX-49–08	1.0 ± 0.0	NA	NA	NA			
LdX-61–02	2.0 ± 0.0	1.89 ± 0.03	0.66 ± 0.01	0.47 ± 0.01			
LdX-61–05	1.86 ± 0.10	1.35 ± 0.07	0.38 ± 0.05	0.24 ± 0.04			
LdX-61–06	1.86 ± 0.10	1.34 ± 0.06	0.38 ± 0.05	0.23 ± 0.04			
LdX-61–09	2.14 ± 0.14	1.37 ± 0.05	0.43 ± 0.05	0.26 ± 0.03			
LdX-61–10	1.0 ± 0.0	NA	NA	NA			
LdX-61–11	1.0 ± 0.0	NA	NA	NA			

*Females*	N_a_	N_e_	H_o_	H_e_	PIC	F_IT_	W_TPM_
LdX-49–01	2.0 ± 0.0	1.54 ± 0.05	0.38 ± 0.03	0.34 ± 0.02	0.289	0.10 ± 0.07	0.199
LdX-49–03	1.0 ± 0.0	NA	NA	NA	NA	NA	NA
LdX-49–05	3.57 ± 0.14	2.84 ± 0.08	0.57 ± 0.04	0.64 ± 0.01	0.624	0.11 ± 0.07	0.075
LdX-49–08	1.0 ± 0.0	NA	NA	NA	NA	NA	NA
LdX-61–02	2.0 ± 0.0	1.88 ± 0.03	0.505 ± 0.02	0.47 ± 0.01	0.369	0.09 ± 0.06	0.051
LdX-61–05	2.0 ± 0.0	1.34 ± 0.04	0.246 ± 0.03	0.24 ± 0.03	0.224	0.02 ± 0.06	0.328
LdX-61–06	2.0 ± 0.0	1.32 ± 0.04	0.198 ± 0.03	0.24 ± 0.02	0.217	0.10 ± 0.09	0.345
LdX-61–09	2.57 ± 0.14	1.48 ± 0.06	0.286 ± 0.02	0.31 ± 0.03	0.290	0.04 ± 0.05	0.297
LdX-61–10	1.0	NA	NA	NA	NA	NA	NA
LdX-61–11	1.0	NA	NA	NA	NA	NA	NA

N_a_ – alleles per locus, N_e_ – effective alleles, I – Shannon’s diversity index, h – haploid genetic diversity, H_o_ – heterozygosity observed, H_e_ – heterozygosity expected, PIC – polymorphic information content, F_IT_ – fixation index, W_TPM_ – *p*-value of Wilcoxon test of heterozygosity excess for two phase model (TPM) of mutation-drift equilibrium. NA – not applicable for monomorphic loci and/or hemizygous males.

**Table 4:** Estimates of genetic diversity parameters of sub-populations (mean ± SD) in 10 loci.

Sub-population							
*Males*	N_a_	N_e_	I	h	P_%_	–	–
Belcice	1.73 ± 0.28	1.36 ± 0.12	0.33 ± 0.10	0.21 ± 0.07	54.6		
Bojanovice	1.73 ± 0.27	1.37 ± 0.14	0.33 ± 0.11	0.21 ± 0.07	54.6		
Ceske Budejovice	1.55 ± 0.21	1.33 ± 0.12	0.29 ± 0.10	0.19 ± 0.07	45.5		
Celakovice	1.64 ± 0.20	1.39 ± 0.14	0.32 ± 0.10	0.21 ± 0.07	54.6		
Chlumin	1.64 ± 0.31	1.32 ± 0.19	0.26 ± 0.12	0.15 ± 0.07	36.4		
Cizov	1.36 ± 0.20	1.23 ± 0.13	0.19 ± 0.10	0.12 ± 0.07	27.3		
Havlickova Borova	1.82 ± 0.30	1.46 ± 0.19	0.37 ± 0.12	0.23 ± 0.07	54.6		
Jamy	1.64 ± 0.20	1.39 ± 0.17	0.31 ± 0.11	0.20 ± 0.07	54.6		
Malonty	1.64 ± 0.20	1.27 ± 0.11	0.26 ± 0.09	0.16 ± 0.06	54.6		
Oblajovice	1.73 ± 0.27	1.40 ± 0.21	0.32 ± 0.12	0.19 ± 0.07	54.6		
Stakcin	1.64 ± 0.20	1.41 ± 0.17	0.33 ± 0.11	0.21 ± 0.07	54.6		
Valecov	1.64 ± 0.20	1.40 ± 0.14	0.33 ± 0.11	0.22 ± 0.07	54.6		
Zabcice	1.64 ± 0.20	1.37 ± 0.17	0.31 ± 0.11	0.19 ± 0.07	54.6		
Zeliv	1.73 ± 0.27	1.31 ± 0.12	0.30 ± 0.10	0.18 ± 0.06	54.6		

*Females*	N_a_	N_e_	I	H_o_	H_e_	F_ST_	W_TPM_
Belcice	1.82 ± 0.30	1.37 ± 0.15	0.33 ± 0.11	0.21 ± 0.08	0.20 ± 0.07	− 0.04 ± 0.10	1.000
Bojanovice	1.73 ± 0.27	1.43 ± 0.18	0.35 ± 0.12	0.16 ± 0.07	0.22 ± 0.07	0.28 ± 0.11	**0.047**
Ceske Budejovice	1.64 ± 0.20	1.38 ± 0.15	0.32 ± 0.10	0.19 ± 0.06	0.20 ± 0.07	0.05 ± 0.06	0.156
Celakovice	1.64 ± 0.20	1.37 ± 0.17	0.31 ± 0.11	0.24 ± 0.08	0.20 ± 0.07	− 0.21 ± 0.02	0.438
Chlumin	1.82 ± 0.30	1.45 ± 0.17	0.37 ± 0.12	0.17 ± 0.05	0.23 ± 0.07	0.20 ± 0.07	0.176
Cizov	1.73 ± 0.24	1.45 ± 0.19	0.35 ± 0.12	0.13 ± 0.05	0.22 ± 0.07	0.42 ± 0.07	0.438
Havlickova Borova	1.64 ± 0.20	1.26 ± 0.11	0.26 ± 0.09	0.18 ± 0.07	0.16 ± 0.06	− 0.16 ± 0.04	0.563
Jamy	1.73 ± 0.24	1.37 ± 0.18	0.31 ± 0.11	0.22 ± 0.08	0.20 ± 0.07	− 0.13 ± 0.05	1.000
Malonty	1.82 ± 0.30	1.46 ± 0.20	0.37 ± 0.12	0.24 ± 0.08	0.23 ± 0.07	− 0.12 ± 0.04	0.156
Oblajovice	1.73 ± 0.27	1.43 ± 0.18	0.35 ± 0.12	0.22 ± 0.08	0.22 ± 0.07	− 0.01 ± 0.06	**0.047**
Stakcin	1.64 ± 0.20	1.41 ± 0.16	0.33 ± 0.11	0.25 ± 0.02	0.21 ± 0.07	− 0.15 ± 0.06	**0.047**
Valecov	1.82 ± 0.30	1.43 ± 0.20	0.34 ± 0.12	0.20 ± 0.07	0.21 ± 0.07	0.01 ± 0.06	0.844
Zabcice	1.82 ± 0.30	1.45 ± 0.20	0.36 ± 0.12	0.21 ± 0.07	0.22 ± 0.07	− 0.01 ± 0.06	0.563
Zeliv	1.82 ± 0.30	1.34 ± 0.19	0.28 ± 0.11	0.16 ± 0.06	0.16 ± 0.07	− 0.04 ± 0.06	0.438

N_a_ – alleles per locus, N_e_ – effective alleles, I – Shannon’s diversity index, h – haploid genetic diversity, P_%_ – percent of polymorphic loci, H_o_ – heterozygosity observed, H_e_ – heterozygosity expected, F_ST_ – fixation index. W_TPM_ – *p*-value of Wilcoxon test of heterozygosity excess for two phase model (TPM) of mutation-drift equilibrium (significant values are highlighted).

**Table 5:** Analysis of molecular variance (AMOVA) with regional effects: 10 loci, 14 sub-populations, 6 regions (Table 1).

Source of variation *Males* (haploid)	df	SS	MS	Variation (Est.)	P_%_	Φ-value	*p*-value
Among regions	5	6.93	1.39	0.001	0.00	0.001 (Φ_RT_)	0.470
Among sub-populations	8	10.83	1.35	0.016	1.00	0.014 (Φ_PR_)	0.203
Within sub-populations	175	200.23	1.14	1.144	99.0	0.014 (Φ_PT_)	0.137
Total	188	217.98		1.161	100.0		

Source of variation *Females* (diploid)	df	SS	MS	Variation (Est.)	P_%_	F-value	*p*-value
Among regions	5	9.65	1.93	0.005	0.00	0.005 (F_RT_)	0.173
Among sub-populations	8	12.64	1.58	0.010	1.00	0.009 (F_SR_)	0.099
Among individuals	214	266.73	1.25	0.080	7.00	0.013 (F_ST_)	0.019*
Within individuals	228	247.50	1.09	1.086	92.0	0.069 (F_IS_)	0.008**
Total	455	536.52		1.181	100.0	0.081 (F_IT_)	0.002**

df – degree of freedom, SS – sum of squares, MS – mean square, P – percent variation, Φ and F – fixation index and its compounds, *or** significant or highly significant genetic differentiation, respectively.

### Ethical approval

All the experimental procedures were conducted in accordance with Czech legislation (Sect. 29 of Act No. 246/1992 Coll. on the protection of animals against cruelty, as amended by Act No. 77/2004 Coll.). We hereby declare that animal handling conducted in our study complies with the relevant European and international guidelines on animal welfare, namely Directive 2010/63/EU on the protection of animals used for scientific purposes and the guidelines and recommendations of the Federation of Laboratory Animal Science Associations. All experimental protocols were approved by the Czech University of Life Sciences Prague, Faculty of Agrobiology, Food and Natural Resources of the Czech Republic and Institutional and National Committees. Adults of CPB were collected in experimental and production potato fields with consent of their owners.

## Results

### Design and general population characteristics of markers

**Figure 3:**
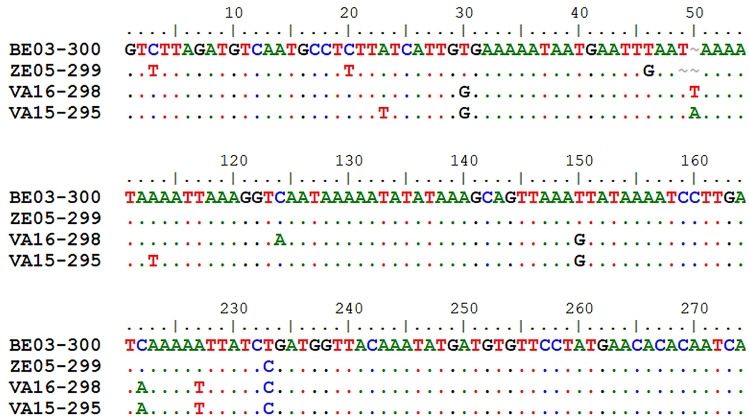
Differences in the sequences of alleles of the locus Ld49_05 (Bioedit version 7.5.3^35^). The names of alleles reflected the sizing using capillary electrophoresis.

From the 21 markers designed, 10 loci (Fig. 2, Table 2) were found suitable for multiplex analysis and six of them were polymorphic in the population sample of 420 CPB adults. The sequencing of amplicons confirmed the identity of loci and their alleles. The sequences of all alleles were submitted to database NCBI GenBank under the following accession numbers: Ld4901 (OR532473–OR532476), Ld4903 (OR532477–OR532478), Ld4905 (OR532461–OR532472), Ld4908 (OR532479–OR532480), Ld6102 (OR532481–OR532484), Ld6505 (OR532485–OR532488), Ld6106 (OR532489–OR532492), Ld6109 (OR532493–OR532498), Ld6110 (OR532499–OR532500) and Ld6111 (OR532501–OR532502). The length polymorphisms of most alleles reflected changes in the number of microsatellite motif repetitions, however, the variability of locus Ld49_05 was enriched by distinctive mutations in sequences around the target motif, which resulted specifically in alleles 298, 299 and 300. The alleles 299 and 300, having motif (TTCA)_4_, showed a similarity of 98%. The more distant alleles 295 and 298 had five repetitions, (TTCA)_5_, combined with two and one, respectively, additional deletions. Several allele-specific substitutions were also observed at this locus (Fig. 3).

The number of alleles and general population characteristics for loci (Table 3) and sub-populations (Table 4) were estimated separately for adult CPB females (N = 231) and males (N = 189). In males, two different alleles per genotype were never detected, which confirmed hemizygous constitution of the males and correct localisation of loci on the chromosome X. Consequently, the genetic diversity of males could only be evaluated using models for haploids and compared to females, the range of population descriptors is reduced. The locus-specific parameters estimated (Table 3) for males and females were generally comparable. All loci show relatively low range of polymorphism, and relatively high fixation (F_IT_). No locus showed a statistically significant heterozygosity excess due to recent bottleneck effect. The diversity parameters across populations were generally homogeneous. In the locality Cizov, however, the low diversity was indicated by Shannon index, h-value, and P_%_ (percent of polymorphic loci) in the group of males, and similarly in H_e_ (heterozygosity expected) in females. The reduced heterozygosity was then reflected in very high value of F_ST_ (0.42) of females. However, the significant heterozygosity excess was found only for populations from Bojanovice, Oblajovice and Stakcin. The analysis of molecular variance (AMOVA) did not detect any regional differences in variability in both groups of males and females. However, the AMOVA indicated significant differentiation of diversity among (F_ST_) and within (F_IS_) individuals, which were consequently reflected in significant F_IT_-value (Table 5).

### Detection of haplotypes in adult CPB

**Figure 4:**
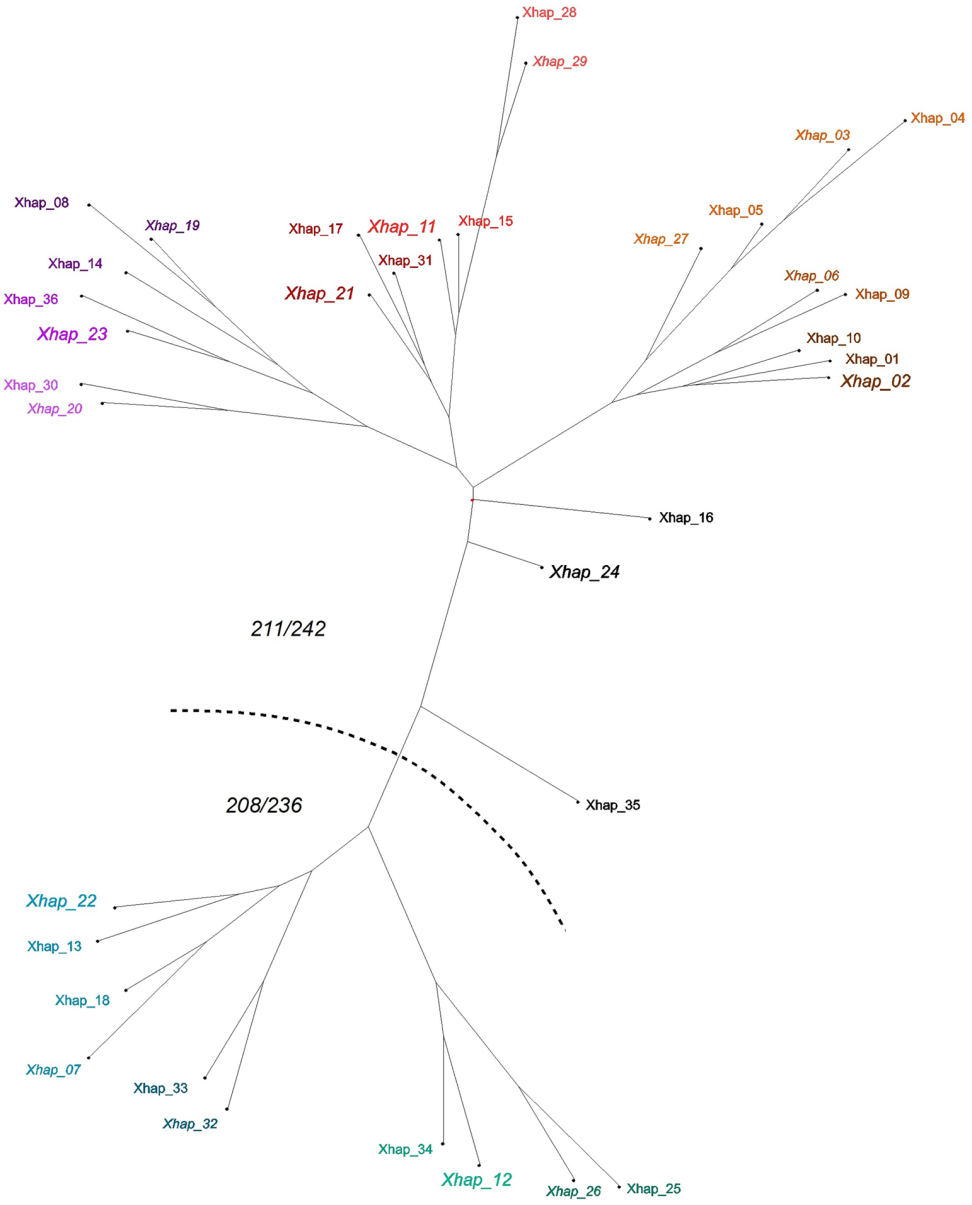
Neighbour joining analysis of X-haplotypes dissimilarity. Different colours and their sub-tones indicate the placement of Xhaps to groups and sub-groups respectively. The most frequent Xhaps are highlighted using italics and magnification. The dashed line indicates two clusters based on differential arrangement of alleles in tightly linked loci Ld6105 and Ld6106.

All mentioned polymorphisms were considered in subsequent analyses of haplotypes. In general, the number of alleles detected across the CPB adult males indicated a potential of 192 X-haplotypes (Xhaps). However, only 36 specific Xhaps (Xhap_01-36) were finally detected in the adult males. Haplotypes and their frequencies are presented in Supplementary Table S1. Genetic similarity of haplotypes, evaluated using neighbour-joining analysis, is shown in Fig. 4. Xhaps were organised in 7 haplotype groups, and each group was divided into sub-groups (from 1 to 4). The sub-groups are clusters of highly similar Xhaps differentiated by a single locus. Xhaps were unevenly distributed; five of the most frequent Xhaps, repeating more than ten times, were detected in 55% of males, 19 repeating up to ten times in 38.7% of males, and the remaining 12 occurred uniquely in 6.3% of males. The most frequent haplotypes within the haplotype groups were considered as a standard, the least frequent haplotypes probably derived from them as recombinations. The pseudo-group of Xhap_24 represents the most common set of three unsortable haplotypes which differed in two loci. Further diversification from this site probably produced two opposite clusters in Fig. 4 characterised by one of the most common and very stable allelic arrangements (211/242 or 208/236) of tightly linked microsatellites Ld6105 and Ld6106. In contrast, the group of Xhap_23, was found as the most complex and variable. The members of its sub-groups were less frequent, and very diversified by various simple crossovers or mutations.

**Figure 5:**
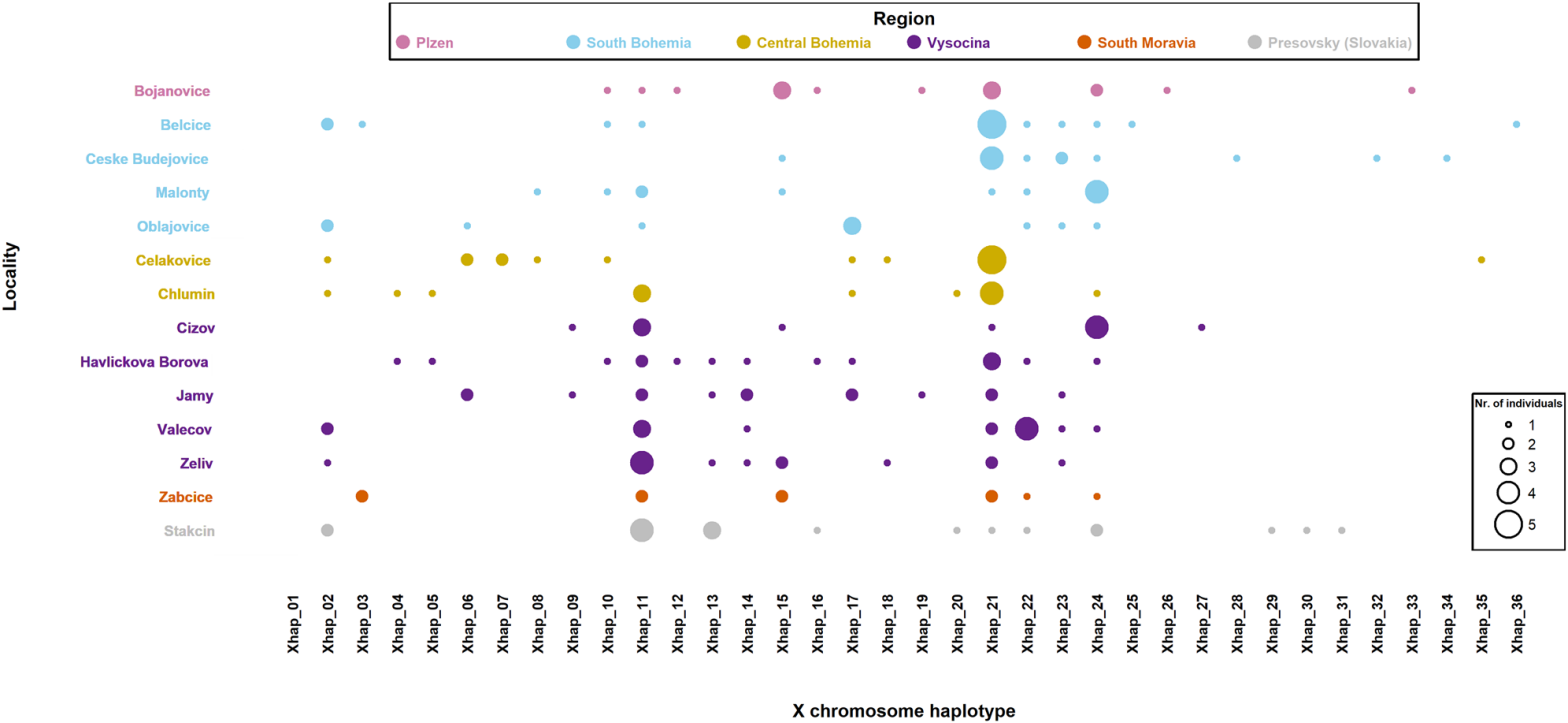
Regional distribution and frequency of the Xhaps. The differential distribution of Xhap_11 and Xhap_21 can be explained by the limited hybridisation of sub-populations in the west–east direction.

The regional distribution of the Xhaps is shown in Fig. 5, where the general frequencies of Xhaps correspond relatively well with their frequencies in the regions. However, a certain geographical trend in the distribution of two haplotypes was found, where the Xhap_21, mostly prevalent in western Czechia, is subsequently almost completely replaced by Xhap_11 in the eastern direction.

### Results of haplotype analysis in clutches

The females TR10-RU14 with their clutches were specifically chosen from a larger group, because they offered a considerable number of larvae (Table 1) and were also heterozygous in various number of loci across both scaffolds (N_hm_ in Table 6). Within the 866 larvae, 13 recombinations of maternal Xhaps were identified, produced by seven ways of crossing-over. The frequency of recombinations was relative to a CPB family, a site, and a type of crossing-over. For example, in the family of TR12, a simple recombination (SCO) between scaffolds 49 and 61 was observed in 37 individuals, when a double crossing-over (DCO) occurred only three times. Twelve of the recombinations were identical with some Xhap detected in the adults and one was an alternative of Xhap_16 with allele 315 instead of allele 318, which belongs to the haplogroup Xhap_24. All the significant results are summarised in Table 6.

**Table 6:** Maternal X-haplotypes and their recombinations observed in progenies.

Mother/progeny	Maternal X-haplotypes	N_hm_	N_sco_	SCO results (freq.)	N_dco_	DCO results (freq.)
TR10	Xhap_02, Xhap_21	2 + 0	1	Xhap_31 (0.008) Xhap_10 (0.008)	0	NA
TR11	Xhap_21, Xhap_25	2 + 4	1	Xhap_02 (0.036) Xhap_34 (0.024)	2	Xhap_31 (0.004) Xhap_26 (0.004) Xhap_22 (0.004)
TR12	Xhap_04, Xhap_34	2 + 3	1	Xhap_25 (0.087) Xhap_36 (0.102)	1	Xhap_26 (0.005) mXhap_16 (0.010)
TR14	Xhap_10, Xhap_21	1 + 0	0	NA	0	NA
RU14	Xhap_15, Xhap_22	1 + 2	1	Xhap_24 (0.053) Xhap_13 (0.067)	0	NA

N_hm_ – number of heterozygous loci of scaffolds 49 and 61 respectively, N_sco_ – number of simple crossovers observed (SCO), N_dco_ – number of double crossovers (DCO), mXhap – modified Xhap.

**Figure 6:**
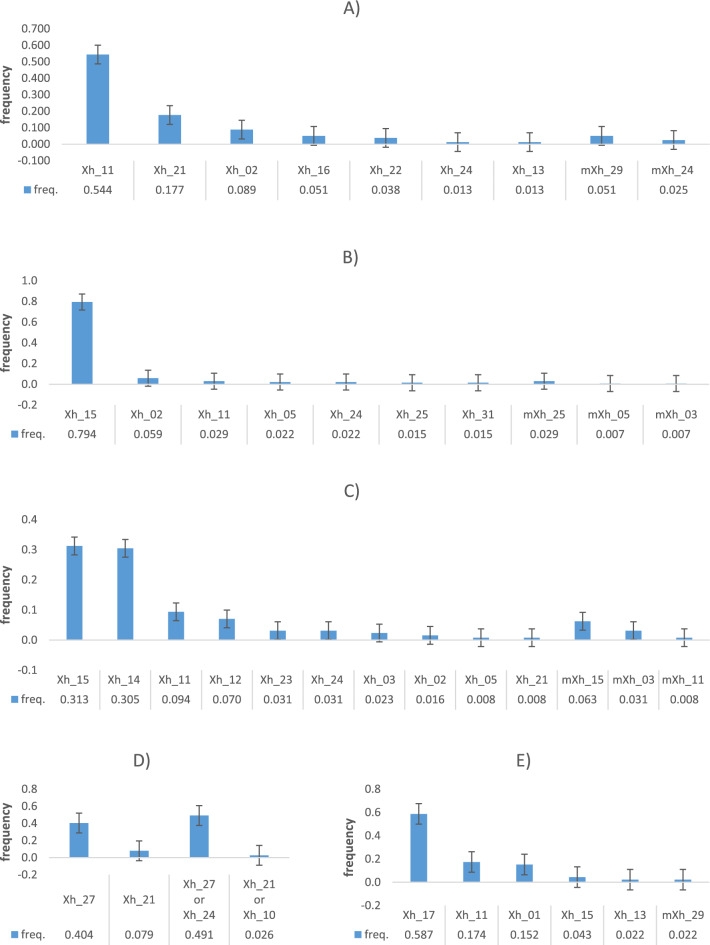
Standard paternal Xhaps (Xh) and their modifications (mXh) in families: **A** TR10, **B** TR11, **C** TR12, **D** TR14, **E** RU14. The bars denote a standard error of 5%.

When analysing the paternal Xhaps in a subset of female-larvae (N = 509), from 6 to 13 different paternal Xhaps were detected, which confirmed polyandry as a common and frequented sexual behaviour of the CPB. Additionally, the analysis identified nine new paternal Xhaps, previously undetected in the adults (Fig. 6). Eight of them were probably produced by SCOs of various Xhaps and one by the DCO of Xhap_29.

## Discussion

### The approach and its role in reaching the research objectives

The samples of individuals were collected in relatively distant fields (minimal distance between localities was 50 km). The system of sampling aided to obtain unrelated samples. It was assumed, that the risk of relatedness during spring is very low, since the initial population consists of very mobile overwintering adults of unknown origin. Especially in localities where the pest occurs frequently, and the density of potato fields is higher.

The analyses of microsatellites are standardly used in CPB to study the general genetic diversity and relatedness of various populations. They can help in identifying the influence of key progenitors or environmental/ecological conditions on population stability and development (i.e. ^1,13^.). Generally, an effective microsatellite analysis uses unlinked markers to prevent an unwanted meiotic co-segregation of alleles. However, we designed the panel of mutually X-linked markers intentionally with the primary objective to utilise stable, nevertheless sufficiently variable, chromosomal segments. This was done to evaluate the number and participation of fathers on half-sibs produced by specific females in polyandric system of CPB reproduction.

Due to the XO sex determination^9,10^, each CPB male has only one copy of chromosome X, which can be identified without any change in all its daughters using a background of known maternal haplotypes. However, a risk of meiotic recombinations between maternal X-haplotypes must be considered. In the design of the experimental background, we faced two challenges: to identify correct unassembled scaffolds of the CPB genome representing the chromosome X; and to select loci enough variable and as tightly linked as possible to reduce a negative influence of meiotic recombination on the experimental results.

To resolve it, we used the evolutionary relatedness and chromosomal homology of CPB with red flour beetle (RFB), and found the unassembled CPB scaffolds 49 and 61, published by i5K Consortium^23^, homologous with the RFB chromosome X assembled by Richards et al.^24^. Recently, Yan et al.^16^ published a chromosomally assembled CPB genome and identified the chromosome 6 with chromosome X. We checked the positions of our loci which were all found in the region between the 52 Mb and the 56 Mb (localisation presented in Table 2) in distances comparable to those shown in Fig. 2. In this region, 90 genes were identified (Supplementary Table S2), where two are tightly linked with microsatellites of group Ld61 and one with loci of group Ld49. This could provide an interesting foundation for future research into the influence of these genes on population dynamics of CPB.

Considering the recombination potential in subjected regions, we observed that the distances of microsatellites did not prevent crossing-overs (Table 6). Some recombinations were observed in families from Travcice and Ruzyne, however, they were too rare and did not disturb the identification of maternal and paternal haplotypes. We presume that the recombinations were more probable resource of variability than mutations, because: i) the changes in haplotypes were relatively frequent (changes occurred in almost 4.2% of maternal haplotypes), ii) we did not observe any mosaicism (heterozygosity) in males, which should be a natural consequence of mutations, iii) the used microsatellites were less variable (from 1 to 4 stable alleles were detected per locus and population) and iv) the microsatellites with tri-, tetra- and penta-nucleotide motifs should be relatively resistant to expansion and contraction. On the other hand, the variability of Ld4905 proves an effect of other mutations (indels) on the allelic diversity.

The unequal frequency of Xhaps could indicate an X-linked bias in fitness of males, while the more frequent haplotypes may represent the most vigorous standard preferred in current environmental conditions. This opportunity is implied by the analysis of bottleneck effects, which pointed at a slightly significant heterozygosity excess in Bojanovice, Oblajovice and Stakcin. The model used for the evaluation should be reliable despite the low number of polymorphic loci^32^. However, the number of females evaluated per sub-population was lesser than the recommended N = 30, and probably insufficient to reach an optimal reliability^32,36^.

### X-haplotypes and their distribution in population and regions

The analysis was performed on a relatively limited number of individuals (N = 420) comprising of 189 random males important especially for the detection of Xhaps and 231 random females important for the unbiased evaluation of genetic diversity, which showed that distribution of the allelic diversity across the Czech population is relatively homogeneous and supports unbiased evaluation of the Xhaps diversity. The sample of experimental adults also showed a general female-biased sex-ratio, as was previously observed^21^ in CPB 1st instar larvae. The 36 Xhaps distributed in adult males with 7 new unique recombinations in larvae represented a reduced part of all possible (N = 192) combinations of alleles. The reduced variability in studied loci could be explained by the relatively short history of the CPB in Czechia, which occurs here since 1945^37^, complemented with a hypothetically low mutational/recombination activity in the chromosome. However, a presence of the gene *lethal 2* (XM_023166821.1) in studied chromosomal region (Table S2) also indicates an early developmental lethality of males, which was well documented for *Drosophila melanogaster*^38^. This might explain both the female-biased sex ratio CPB and an evolutional dynamic of Xhaps. Because the pure haplotypes could only be identified in hemizygous males and homozygous females, the potential lethal combinations, maintained in heterozygous females, were undetected.

The results also showed an inequal geographical (regional) distribution of the Xhaps. Figure 2 presents a relatively undisturbed migration corridor alongside the Vltava River. Despite the geographical distance between Ceske Budejovice (South Bohemia) and Chlumin (Central Bohemia), which exceeds 200 km, the frequencies of more dominant Xhaps are similar (Fig. 5). However, the Fig. 5 also clearly shows a gradient in frequency of Xhap_21 (highly frequented in South and Central Bohemia regions) and Xhap_11 (almost exclusive for the Vysocina region and Slovakia). The localities Belcice (South Bohemia) and Zeliv (Vysocina) are distant about 120 km, and we see a drastic change in the composition of haplotypes with an increase in frequency of Xhap_11. The in between locality of Oblajovice is instead characterised by a higher occurrence of Xhap_17, which belongs to the Xhap_21 group, and which may represent an acceptable genetic bridge between Xhap_21 and Xhap_11. It indicates a barrier disturbing migration between the landscape around the Vltava River and the Bohemian-Moravian Highlands (Fig. 2). The environmental difference which can explain this barrier could be the altitude. The Bohemian-Moravian Highlands (Vysocina region), with an altitude from 450 to 700 m. a. s. l., represents the most traditional area for potatoes growing in Czechia. Although the negative impact of CPB has relatively increased here within the last 30 years, beetles are still univoltine here and dependent on the weather conditions in winter and spring^17^. This causes a delay in development for two weeks, which limits a migration in the west-eastern orientation. Compared with highlands, the conditions of landscape alongside large rivers of Czechia are generally warmer due to a lower altitude of up to 400 m. a. s. l. The potatoes emerge here earlier, and CPB are generally bivoltine. Moreover, more homogeneous microclimate in lowlands can support migration on larger distances and mixing of regional sub-populations in a north–south direction. The situation should change in future, because Kocmánková et al.^39^ predicted climatic changes in Czechia, that should support a bivoltinism of CPB by 43% and rise of CPB to higher altitudes. However, further research of presented genetic indicators is needed to confirm a positive selection of Xhaps linked with environmental adaptation, because the AMOVA did not confirm a significant regional nor population differentiation in Xhaps diversity.

### The number and proportionality of paternal haplotypes in half-sib progenies

Polyandry is a sexual behaviour, which maintains both the genetic diversity of the population and the fitness of progenies. The impact on the oviposition and fitness of the female and its progeny were, for example, experimentally confirmed^40^ for horned flour beetle. In the CPB, polyandry was confirmed as a common reproduction strategy^21^; the reality in terms of the number of participating males and their contribution to the progeny genetic diversity, however, remained unknown. The X-haplotype analysis is used to clarify these problems in present work. Because the male-larvae represent the maternal X-haplotypes, we analysed the haplotype of all available female-larvae in each clutch to estimate the paternal contribution. On average, 8.4 ± 3.5 males contributed on X-haplotypes per clutch. However, this number could be an underestimation because of the high frequency of some haplotypes shared by males in population. Boiteau^41^ proved that CPB females require at least 3 copulations with different males to full fill their oviposition capacity. The observations herein have therefore been somewhat surprising and yet, convincing proof of almost unlimited promiscuity and/or absence of any sexual preferences or selection in CPB. The last-male precedence has been shown in CPB by previous works (e.g. ^11,12^.). Our results also confirmed that last males mostly contributed to the fertilization of eggs (Fig. 6). This was due to an exclusive haplotype occurring in 4 of the 5 evaluated families in a frequency higher than 50%. While this suggests that either sperm of the last male is fresher or the first to be used, in our temporary lab cultures, the ovipositions were mostly discontinuous and produced from 2 to 8 sub-clutches. Keeping the integrity of the sub-clutches and analysing them separately (data not shown), we found that the paternal haplotypes were distributed proportionally across all sub-clutches. This indicates that the sperms in spermatheca are rather effectively mixed with sperms of previous males reduced by previous oviposition. This corresponds to the results of experiment by Roderick et al.^11^, although the duration and number of copulations of our females were unknown. There is also still a question if some sperms from autumnal copulations effectively participated in offspring.

In conclusion, the presented research contributed to characterisation of the genetic diversity in adult CPB populations and better understanding of the polyandrous reproductive strategy of CPB. Some observations indicated the need for further research of X-linked environmental selection and bias in fitness of CPB males.

### Supplementary Information

Supplementary Table S1.

Supplementary Table S2.

## Data Availability

The datasets generated and analyzed during the current study are available from the corresponding author upon reasonable request.
