# Supplementary material for: Investigation of genetic diversity and polyandry of Leptinotarsa decemlineata using X-linked microsatellite markers
Source: Sci Rep. 2023 Dec 11;13:21887. doi: 10.1038/s41598-023-49002-7 (PMC10713635; doi:10.1038/s41598-023-49002-7)
Supplement: Supplementary file 1 — Supplementary Table S1. [file 41598_2023_49002_MOESM1_ESM.pdf]

Supplementary Table S1. X-haplotypes detected in the population sample of adult CPB males (N=189). Names respect the order in which the haplotypes were organised by clonal analysis in GenAlEx. X-haplotypes are organised in groups (families) indicated by neighbour joining processed in Darwin 6.0 (Fig. 4)

**X-hapl. family**	**X-haplotype**	**Ld49_01**	**Ld49_05**	**Ld61_02**	**Ld61_05**	**Ld61_06**	**Ld61_09**	**Freq.**
Xhap_12	*Xhap_12*	*147*	*295*	*252*	*208*	*236*	*309*	*0.011*
	Xhap_34	147	300	252	208	236	309	0.005
	Xhap_25	144	298	252	208	236	309	0.005
	Xhap_26	144	300	252	208	236	309	0.005
Xhap_22	*Xhap_22*	*147*	*300*	*252*	*208*	*236*	*318*	*0.058*
	Xhap_13	147	295	252	208	236	318	0.032
	Xhap_18	147	299	252	208	236	318	0.011
	Xhap_07	144	299	252	208	236	318	0.011
	Xhap_32	147	299	244	208	236	318	0.005
	Xhap_33	147	300	244	208	236	318	0.005
Xhap_24	*Xhap_24*	*147*	*300*	*252*	*211*	*242*	*318*	*0.101*
	Xhap_35	147	300	252	211	236	318	0.005
	Xhap_16	147	298	252	211	242	318	0.016
Xhap_21	*Xhap_21*	*147*	*300*	*244*	*211*	*242*	*318*	*0.185*
	Xhap_17	147	299	244	211	242	318	0.042
	Xhap_31	147	298	244	211	242	318	0.005
Xhap_23	*Xhap_23*	*147*	*300*	*252*	*211*	*242*	*309*	*0.037*
	Xhap_36	147	300	252	211	242	315	0.005
	Xhap_08	144	299	252	211	242	309	0.011
	Xhap_19	147	299	252	211	242	309	0.011
	Xhap_14	147	295	252	211	242	309	0.026
	Xhap_20	147	300	244	211	242	309	0.011
	Xhap_30	147	298	244	211	242	309	0.005
Xhap_02	*Xhap_02*	*144*	*298*	*244*	*211*	*242*	*318*	*0.058*
	Xhap_10	144	300	244	211	242	318	0.026
	Xhap_01	144	295	244	211	242	318	0.016
	Xhap_06	144	299	244	211	242	318	0.026
	Xhap_09	144	299	252	211	242	318	0.011
	Xhap_03	144	298	252	211	242	309	0.016
	Xhap_04	144	298	252	211	242	315	0.011
	Xhap_05	144	298	252	211	242	318	0.011
	Xhap_27	144	300	252	211	242	318	0.005
Xhap_11	*Xhap_11*	*147*	*295*	*244*	*211*	*242*	*318*	*0.148*
	Xhap_28	147	295	244	208	242	318	0.005
	Xhap_15	147	295	252	211	242	318	0.053
	Xhap_29	147	295	252	208	242	318	0.005
