# Supplementary material for: Investigation of genetic diversity and polyandry of Leptinotarsa decemlineata using X-linked microsatellite markers
Source: Sci Rep. 2023 Dec 11;13:21887. doi: 10.1038/s41598-023-49002-7 (PMC10713635; doi:10.1038/s41598-023-49002-7)
Supplement: Supplementary file 2 — Supplementary Table S2. [file 41598_2023_49002_MOESM2_ESM.pdf]

Table S2: Positions of the protein-coding genes localised on chromosome 6 (chromosome X) in region of interest. The genes present in the clusters of markers Ld49 and Ld61 are labelled by green and blue colour respectively.

**Gene (CDS)**	**NCBI accession**	**Scaffold accession**	**Locus on chr. X (bp)**
sphingomyelin phosphodiesterase-like	XM_023167259.1	NW_019291515.1	51738136 - 51798719
glucose-6-phosphate 1-dehydrogenase-like	XM_023167230.1	NW_019291500.1	51830624 - 51845045
isocitrate dehydrogenase [NAD] subunit gamma, mitochondrial-like	XM_023167229.1	NW_019291500.1	51857652 - 51845484
actin-related protein 6-like	XM_023167235.1	NW_019291500.1	51862660 - 51864602
probable DNA mismatch repair protein Msh6	XM_023167236.1	NW_019291500.1	51885630 - 51872141
suppressor protein SRP40-like	XM_023167228.1	NW_019291500.1	51886430 - 51887807
ras-like protein 3	XM_023167232.1	NW_019291500.1	51891016 - 51920582
ionotropic receptor 21a-like	XM_023172561.1	NW_019296338.1	52076494 - 52088651
protein let-756	XM_023157644.1	NW_019289443.1	52744627 - 52525199
rho guanine nucleotide exchange factor 17 isoform X3	XM_023157670.1	NW_019289443.1	52977995 - 52890435
rho guanine nucleotide exchange factor 17 isoform X2	XM_023157662.1	NW_019289443.1	52977995 - 52890435
rho guanine nucleotide exchange factor 17 isoform X1	XM_023157656.1	NW_019289443.1	52977995 - 52890435
protein tipE	XM_023157692.1	NW_019289443.1	53029807 - 53028096
uncharacterized protein LOC111503383	XM_023157680.1	NW_019289443.1	53029823 - 53041513
protein tipE	XM_023157702.1	NW_019289443.1	53037810 - 53041513
uncharacterized protein LOC111503475	XM_023157776.1	NW_019289443.1	53056155 - 53074687
transcription initiation factor TFIID subunit 7	XM_023157712.1	NW_019289443.1	53097321 - 53092411
nucleolar transcription factor 1-A-like	XM_023157788.1	NW_019289443.1	53119361 - 53106491
sodium/hydrogen exchanger 7 isoforms X1	XM_023157718.1	NW_019289443.1	53125767 - 53152916
sodium/hydrogen exchanger 7 isoform X4	XM_023157743.1	NW_019289443.1	53125767 - 53152916
sodium/hydrogen exchanger 7 isoform X3	XM_023157735.1	NW_019289443.1	53125767 - 53152916
sodium/hydrogen exchanger 7 isoform X6	XM_023157754.1	NW_019289443.1	53125767 - 53152916
sodium/hydrogen exchanger 7 isoform X2	XM_023157725.1	NW_019289443.1	53125767 - 53152916
sodium/hydrogen exchanger 7 isoform X5	XM_023157750.1	NW_019289443.1	53125767 - 53152916
ELAV-like protein 3 isoform X1	XM_023173461.1	NW_019289520.1	53212040 - 53160086
ELAV-like protein 3 isoform X2	XM_023173462.1	NW_019289520.1	53220952 - 53160086
ELAV-like protein 3 isoform X1	XM_023173459.1	NW_019289520.1	53220952 - 53160086
ELAV-like protein 3 isoform X1	XM_023173460.1	NW_019289520.1	53244948 - 53160086
cytosolic carboxypeptidase 6	XM_023173454.1	NW_019289520.1	53260765 - 53282542

nipped-B-like protein	XM_023173455.1	NW_019289520.1	53293975 - 53311192
uncharacterized protein LOC111517343 isoform X1	XM_023173463.1	NW_019289520.1	53335723 - 53334526
uncharacterized protein LOC111517343 isoform X2	XM_023173464.1	NW_019289520.1	53335723 - 53334526
uncharacterized protein LOC111517340	XM_023173456.1	NW_019289520.1	53374479 - 53339818
uncharacterized protein LOC111517344 isoform X1	XM_023173465.1	NW_019289520.1	53378905 - 53520214
uncharacterized protein LOC111517344 isoform X1	XM_023173467.1	NW_019289520.1	53389287 - 53520214
uncharacterized protein LOC111517344 isoform X2	XM_023173468.1	NW_019289520.1	53492937 - 53520214
uncharacterized protein LOC111517344 isoform X3	XM_023173469.1	NW_019289520.1	53492937 - 53520214
uncharacterized protein LOC111517344 isoform X3	XM_023173470.1	NW_019289520.1	53493634 - 53520214
protein hook	XM_023173458.1	NW_019289520.1	53529251 - 53525303
TBC1 domain family member 22B	XM_023173471.1	NW_019289520.1	53532298 - 53534030
microtubule-associated serine/threonine-protein kinase 4	XM_023157024.1	NW_019289440.1	53597457 - 53611370
protein trachealess isoform X4	XM_023157070.1	NW_019289440.1	53689046 - 53621618
protein trachealess isoform X3	XM_023157061.1	NW_019289440.1	53689047 - 53621618
protein trachealess isoform X2	XM_023157052.1	NW_019289440.1	54027653 - 53621618
protein trachealess isoform X1	XM_023157045.1	NW_019289440.1	54027653 - 53621618
uncharacterized protein LOC111502899	XM_023157081.1	NW_019289440.1	54087401 - 54044898
uncharacterized protein LOC111502866	XM_023157034.1	NW_019289440.1	54227821 - 54090074
SLIT-ROBO Rho GTPase-activating protein 1-like	XM_023166822.1	NW_019291359.1	54386859 - 54407302
NAD-dependent protein deacylase-like	XM_023166823.1	NW_019291359.1	54414134 - 54440944
protein lethal (2) essential for life-like	XM_023166821.1	NW_019291359.1	54417223 - 54418099
steroid receptor seven-up, isoforms B/C isoform X1	XM_023162397.1	NW_019290333.1	54709697 - 54846789
steroid receptor seven-up, isoforms B/C isoform X2	XM_023162398.1	NW_019290333.1	54709699 - 54846789
adult-specific cuticular protein ACP-20-like	XM_023164909.1	NW_019290832.1	54938317 - 54937943
succinate-CoA ligase [ADP/GDP-forming] subunit alpha, mitochondrial	XM_023172475.1	NW_019296108.1	55095764 - 55092062
succinate-CoA ligase [ADP/GDP-forming] subunit alpha, mitochondrial-like	XM_023163452.1	NW_019290542.1	55104972 - 55095764
mannose-1-phosphate guanyltransferase beta	XM_023163450.1	NW_019290542.1	55108001 - 55110732
mannose-1-phosphate guanyltransferase beta	XM_023163451.1	NW_019290542.1	55108276 - 55110734
mitochondrial folate transporter/carrier isoform X1	XM_023163447.1	NW_019290542.1	55115297 - 55132101
mitochondrial folate transporter/carrier isoform X2	XM_023163448.1	NW_019290542.1	55115343 - 55132101
transcription elongation factor SPT4	XM_023163445.1	NW_019290542.1	55139142 - 55137286

uncharacterized protein LOC111508026	XM_023163444.1	NW_019290542.1	55140032 - 55169094
latrophilin Cirl	XM_023162070.1	NW_019289463.1	56114394 - 56488179
uncharacterized protein LOC111506921	XM_023162145.1	NW_019289463.1	56493733 - 56505047
uncharacterized protein LOC111506863	XM_023162080.1	NW_019289463.1	56507263 - 55143279
congested-like trachea protein	XM_023162099.1	NW_019289463.1	56538896 - 56515733
interferon-inducible double-stranded RNA-dependent protein kinase activator A-like	XM_023162120.1	NW_019289463.1	56544319 - 56552462
leucine-rich repeat-containing protein 1	XM_023162110.1	NW_019289463.1	56570320 - 56557753
venom protease-like isoform X2	XM_023162136.1	NW_019289463.1	56571503 - 56598028
venom protease-like isoform X1	XM_023162128.1	NW_019289463.1	56571631 - 56598028
venom protease-like	XM_023162043.1	NW_019289463.1	56604330 - 56647686
NGFI-A-binding protein homolog	XM_023162053.1	NW_019289463.1	56652531 - 56650581
succinate--CoA ligase [GDP-forming] subunit beta, mitochondrial	XM_023166693.1	NW_019291319.1	56849858 - 56847422
LOW QUALITY PROTEIN: IQ motif and SEC7 domain-containing protein 1	XM_023173535.1	NW_019289524.1	56870544 - 56958568
microprocessor complex subunit DGCR8-like	XM_023173534.1	NW_019289524.1	56965070 - 56966053
microprocessor complex subunit DGCR8-like	XM_023173536.1	NW_019289524.1	56976848 - 56969635
filamin-A-like	XM_023173542.1	NW_019289524.1	56992797 - 57048084
filamin-A isoform X1	XM_023173538.1	NW_019289524.1	57098547 - 57197770
filamin-A isoform X3	XM_023173540.1	NW_019289524.1	57119605 - 57197770
filamin-A isoform X2	XM_023173539.1	NW_019289524.1	57158708 - 57197770
filamin-A isoform X3	XM_023173541.1	NW_019289524.1	57158708 - 57197770
cholinesterase	XM_023173549.1	NW_019289524.1	57201699 - 57217561
neurogenic protein big brain	XM_023173543.1	NW_019289524.1	57264550 - 57222271
polyadenylate-binding protein 4-like	XM_023173544.1	NW_019289524.1	57278774 - 57293498
C2 domain-containing protein 5 isoform X1	XM_023173546.1	NW_019289524.1	57295215 - 57319071
C2 domain-containing protein 5 isoform X2	XM_023173547.1	NW_019289524.1	57295299 - 57319071
GILT-like protein 1	XM_023173548.1	NW_019289524.1	57338510 - 57330450
facilitated trehalose transporter Tret1-like	XM_023173533.1	NW_019289524.1	57416780 - 57424381
